# Infracyanine Green vs. Brilliant Blue G in Inverted Flap Surgery for Large Macular Holes: A Long-Term Swept-Source OCT Analysis

**DOI:** 10.3390/medicina56010043

**Published:** 2020-01-20

**Authors:** Salvatore Cillino, Massimo Castellucci, Giovanni Cillino, Valentina Sunseri, Costanza Novara, Francesco Di Pace, Maria Vadalà, Vincenza Bonfiglio, Alessandra Casuccio

**Affiliations:** 1 Department of Biomedicine, Neurosciences and Advanced Diagnostic, Ophthalmology Section, University of Palermo, 90127 Palermo, Italy; massimo.castellucci@gmail.com (M.C.); giovannicillino@alice.it (G.C.); valentinasunseri@hotmail.it (V.S.); costanza.novara88@gmail.com (C.N.); frdipace@tiscali.it (F.D.P.); maria.vadala@unipa.it (M.V.); 2 Department of Ophthalmology, University of Catania, 95123 Catania, Italy; enzabonfiglio@gmail.com; 3 Department of Health Promotion, Mother Child Care, Internal Medicine and of Excellence, University of Palermo, 90127 Palermo, Italy; alessandra.casuccio@unipa.it

**Keywords:** Infracyanine green, brilliant blue G, inverted flap surgery, large macular hole, swept-source OCT

## Abstract

*Background and Objectives:* To compare the long-term toxicity of infracyanine green (IFCG) to brilliant blue G (BBG) in inverted internal limiting membrane flap surgery (I-ILMFS) for large, full-thickness macular holes (FTMHs). *Materials and Methods:* Prospective randomized study including 39 eyes with ≥ 400 µm idiopathic FTMH who underwent I-ILMFS with either IFCG or BBG. Postoperative 6- and 12-month corrected distance visual acuity (CDVA), closure rate, and swept-source optical coherence tomography parameters, including ellipsoid zone (EZ) and external limiting membrane (ELM) mean defect length, central foveal thicknesses (CFT), parafoveal macular thickness (MT), ganglion cells and inner plexiform layer (GCL++) thickness, and peripapillary nerve fiber layer (pRNFL) thickness, were compared. *Results:* Nineteen eyes were included in the IFCG group and 20 eyes in the BBG group. In all cases a FTMH closure was found. CDVA improved at 6 and 12 months in both groups (*p* < 0.0005); the increase at 12 months was greater in the BBG group (p = 0.036). EZ and ELM defects did not differ between groups at either follow-up time. CFT at 12 months was greater in the BBG group (*p* = 0.041). A 12-months compared to 6-months MT decrease was present in both groups (*p* < 0.01). The GCL++ superior inner sector was thicker in the BBG group at 12 months (*p* = 0.036), as were the superior outer sector (*p* = 0.039 and *p* = 0.027 at 6 and 12 months, respectively) and inferior outer sector (*p* = 0.011 and *p* = 0.009 at 6 and 12 months, respectively). *Conclusion:* In our study BBG in I-ILMFS exhibits better long-term CDVA and retinal thickness than does IFCG, suggesting a lesser toxicity from BBG. These findings support the use of BBG over IFCG in I-ILMFS.

## 1. Introduction

A low success rate has been reported when dealing with surgical repair of large, full thickness macular holes (FTMHs), with up to 44% remaining open when the minimum diameter exceeded 500 µm, high frequency of reoperations, and a final visual acuity less than 0.2 logMAR [1,2].

In 2010, Michalewska et al. [3] reported a 98% closure rate using a new surgical approach to large FTMHs, called the inverted inner limiting membrane (ILM) flap surgery (I-ILMFS), where a remnant of ILM left attached to the margins of the hole is inverted and used to fill and cover the hole. Subsequently, both Michalewska et al. and others reported similar closure rates with the same technique on myopic macular holes, either simple or associated with retinal detachment or macular schisis [4,5,6,7].

Various vital dyes have been used during I-ILMFS to stain the ILM and make its removal and trimming easier. These include green-staining cyanine dyes such as indocyanine green (ICG) and infracyanine green (IFCG), which are used off-label in a number of vitreoretinal interface disorders, and blue-staining azo and arylmethane dyes such as trypan blue, brilliant blue G (BBG), and bromophenol blue [8,9,10].

ICG, with its affinity for extracellular matrix components of the ILM such as collagen and fibronectin, had been used for macular hole treatment, since 2000 [11,12]. However, since 2001, experimental and clinical reports have stressed ICG’s toxicity to the retinal ganglion cells and pigment epithelium (RPE), with atrophy of the latter. This, along with worse visual outcomes and visual field defects have substantially decreased its use [13,14,15,16,17,18].

IFCG shares ICG’s affinity for ILM and enhanced visualization, but with less toxic effects due to the absence of sodium iodine, iso-osmolarity in 5% glucose solution, and different light-absorbing properties [8,19,20,21,22,23,24]. Yet, other authors claim risk and evidence of chronic cellular retinal damage even with IFCG due to long intracellular persistence in RPE cells by phagocytosis [18,24,25,26], and direct experimental toxicity on glial cells [18].

Blue-staining dyes are generally regarded as less toxic than green staining ones, especially ICG, based on laboratory data and better postoperative visual acuity, visual fields, microperimetry, retinal microstructure, and multifocal electroretinogram (ERG) in some studies [10,27,28]. Optical coherence tomography (OCT) analysis of central foveal thickness (CFT) and length of the defect of the photoreceptor inner segment/outer segment junction (IS/OS) indicate better outcomes with BBG than ICG in traditional FTMH surgery [29]. However, some experimental and clinical studies indicate a degree of toxicity from blue dyes in vitreoretinal interface and traditional macular hole surgery [24,30,31,32]. Studies using spectral-domain (SD) OCT analysis point out postoperative thinning of the inner retinal layers after BBG-stained ILM peeling, mainly of the temporal macular ganglion cell and inner plexiform layer (GCL++) [30,31,32,33]. This seems indeed related surgical trauma and/or temporal thinning resulting from post-surgical nasal displacement of the fovea, without agreement on the influence of blue dye toxicity [32,33].

Considering the still-frequent use of cyanine dyes in I-ILMFS, especially in eastern Asiatic countries [6,34], and the above, sometimes-conflicting data, we prospectively compared BBG and IFCG in I-ILMFS. Though ICG is still more widely used, IFCG in low concentration could be safer because of its lower toxicity.

To the best of our knowledge, ours is the first study to directly compare I-ILMFS results with two different dyes. We evaluate long-term visual acuity and swept-source OCT (SS-OCT) parameters such as morphology and thickness of macular outer and inner tissue layers and peripapillary nerve fiber layer (pRNFL).

## 2. Materials and Methods

The protocol of this prospective randomized study was approved by the Ethics Committee of the University of Palermo, Italy (R.B. approval No. 11/2016, date of approval 14.1.2016. trial is registered with NCT03946267). From November 2015 to January 2018, all phakic or pseudophakic patients affected with large idiopathic FTMH, with a minimum preoperative diameter of 400 µm, were screened at the Department of Ophthalmology of the University of Palermo, Italy. Written informed consent was obtained from all subjects prior to enrollment. Symptom duration before surgery was noted.

Exclusion criteria were history of chorioretinal disease other than FTMH, myopia > 3 diopters, history of glaucoma, previous trauma or ocular surgery other than cataract extraction, or conditions affecting visual acuity except cataract.

Patients were assigned to undergo vitrectomy with IFCG or BBG using the sealed-envelope randomization technique based on the patients’ surgical chart number, as previously described by Cillino et al. [35]. Phakic subjects with both clinically significant cataracts and subclinical lens opacities were operated on at the time of vitrectomy to avoid postoperative influence on visual acuity due to opacity increase. Investigators involved in clinical data collection and measurement of outcome variables were not directly involved in the patients’ surgery and were masked to the randomization process. Corrected distance visual acuity (CDVA), complete ophthalmic examination, and dilated OCT examination were analyzed at baseline and at 6 and 12 months postoperatively. The randomization code was maintained only at the central data facility and was not broken until all data analysis was complete.

### 2.1. Surgical Procedure

All surgeries were performed by one of two experienced surgeons (S.C. or G.C.). In phakic eyes, immediately before vitrectomy a standard phacoemulsification was performed through a superotemporal or superonasal 2.4-mm near-clear corneal tunnel incision. The implantation of an Acrysof IQ Monofocal intraocular lens (IOL) was performed using a Monarch II or an UltraSert Pre-Loaded Delivery System (Alcon Italia SpA, Milan, Italy). The surgical wound was closed by single stitch 10-0 nylon suture. In all cases, a sutureless 25-gauge, 3-port pars plana vitrectomy (PPV) (Constellation, Alcon Laboratories, Fort Worth, Texas, USA) was performed with both non-contact viewing system (Resight 700, Carl Zeiss Meditec, Jena, Germany) and contact macular lens (Grieshaber disposable (DSP) Aspheric Macular lens, Alcon Laboratories, Fort Worth, Texas, USA). After removal of core and posterior cortical vitreous and hyaloid stained with 0.2 mL triamcinolone acetonide (40 mg/mL; Kenacort, Bristol-Myers Squibb, Anagni, Italy), on the basis of the previous randomization, the ILM was stained with IFCG or BBG. In IFCG subjects, 25 mg IFCG (SERB Laboratories, Paris, France) was dissolved in 10 mL 5% glucose solution. This solution was further diluted 1:4 with balanced salt solution to obtain a final IFCG concentration of 0.5 mg/mL (0.05%, 308 mOsm), 0.2 mL of which were slowly injected over the macular area with the infusion line closed, and carefully washed out and aspirated from vitreous cavity after 45 s. In BBG subjects, 0.1 to 0.2 mL BBG (Brilliant Peel, Fluoron GmbH, Ulm, Germany) at a concentration of 0.25 mg/mL (0.025%) were similarly injected, maintained for 60 s, and carefully removed [26,35]. The ILM was peeled off with ILM forceps, usually beginning near the inferior or superior vascular arcade, at least two disk diameters from the macular hole, in a circular manner. The peeling was extended up to the edges of the macular hole, the wide ILM flap obtained reduced by trimming with the vitreous cutter, and the annular remnant of ILM hinged to the hole’s edge was gently inverted upside down facing the RPE. Therefore, the hole was covered with usually more layers of inverted ILM. Attention was paid to avoid insertion and filling of the hole volume with ILM. Fluid-air exchange was performed at very low intraocular pressure with the backflush needle positioned at least one disk diameter from the hole to avoid flap eversion. At the end of the surgery, air was exchanged with a 20% sulfur hexafluoride (SF_6_) gas mixture and patients were instructed to maintain a prone position for at least 3 days after surgery. All patients received topical ofloxacin (Exocin, Allergan SpA, Rome, Italy) for 7 days and tobramycin and dexamethasone ophthalmic suspension (Tobradex, Alcon Italia SpA) for 4 weeks postoperatively, with dose tapering.

### 2.2. Outcome Measures

Outcomes, recorded and analyzed at baseline, after 6 months and 12 months, were the postoperative CDVA, closure rate, foveal morphology, and length of the defect of the IS/OS (ellipsoid zone, EZ) and of the external limiting membrane (ELM). Moreover, the postoperative thickness maps of the following layers were compared: central fovea (CFT), parafoveal macula (macular thickness, MT), i.e., layer between the inner limiting membrane (ILM) and the outer segment/inner surface of the RPE boundaries, parafoveal and perifoveal GCL++, between the ILM and the inner plexiform/inner nuclear layer boundaries, and pRNFL. CDVA was measured in logMAR notation using ETDRS charts (CC-100XP LCD System for Early treatment diabetic retinopathy study (ETDRS) chart display; Topcon Europe BV, Milano, Italy) at 4 m.

Minimum hole width not less than 400 µm, measured by averaging the vertical and horizontal diameters at the narrowest hole point as lines drawn roughly parallel to the RPE [36], was verified using the manual caliper function of a SD-OCT machine (Topcon 3DOCT-2000 FA Plus, Topcon Italia, Milan, Italy) or a SS-OCT machine (Topcon DRI OCT Triton Series; Topcon Italia, Milan, Italy) on the radial-scan, due to the unavailability of the SS-OCT at the beginning of the study in Italy. The first 5 patients in the IFCG group and 6 patients in the BBG group were analyzed by SD-OCT. The SD- or SS-OCT imaging analysis served also to determine the base diameters of the FTMHs. For simplification purposes, we assumed the EZ and ELM preoperative defects to approximate the base diameter of the hole.

The successful hole closure and configuration, and postoperative sizes of EZ and ELM defects, were assessed by exclusive use of the SS-OCT. In detail, the scan protocol comprised a 12 × 9-mm-wide, three-dimensional (3D) volumetric scan consisting of 512 × 256 A-scans and radial-scan consisting of 12 6-mm-long lines. Both the radial and the 3D scans have been used and matched among them using the “COMPARE” function of the SS-OCT, which allowed the examiner to evaluate the two scans at the same time on the monitor and to move the cursor through the various B-scan. In this way the examiner, before the evaluation, was always sure that the center of the fovea was scanned and stayed in the middle of the B-scan itself. The 3D macular scan images were used to determine the foveal configuration following hole closure and the thickness of different retinal layers, whereas the radial-scan images were used to measure the hole diameter and defects in both the EZ and ELM. The foveal closure configuration included U-shaped, V-shaped, and W-shaped cases [2,37].

Auto-segmentation software (Topcon Advanced Boundary Software) was employed to measure postoperatively by SS-OCT the thickness of the above-described macular and peripapillary layers. The thickness map of each retinal layer was measured around the fovea, and data were grouped as nine macular quadrants within three concentric grids of 1-, 3- and 6-mm diameter, as defined by the ETDRS [38,39]. The layers’ thickness measurements were therefore recorded and displayed as the mean and standard deviation for each of the nine quadrants of the ETDRS grid of IFCG patients and BBG patients separately.

The average thickness in the central 1-mm-diameter circle (C1) represented the CFT. The second 3-mm grid subdivided into superior (S3), nasal (N3), inferior (I3) and temporal (T3) inner macula areas, was used for the MT analysis. Both the second and the third 6-mm grid, the latter composed by the superior (S6), nasal (N6), inferior (I6) and temporal (T6) outer macula areas, were included in the para- and perifoveal GCL++ thickness analysis. A mean thickness was also calculated in the four peripapillary (pRNFL) sectors, i.e., superior (pS), nasal (pN), inferior (pI) and temporal (pT). OCT examination contemplated multiple measurements to obtain an image with quality index above 50.0.

### 2.3. Statistical Analysis

Statistical analysis of quantitative data, including descriptive statistics, was performed for all items. All continuous data are expressed as a mean ± standard deviation. All visual acuity results are presented in logMAR. Univariate analysis of variance was used for parametric analysis. Discrete variables were analyzed using the chi-square test and Fisher exact test, as needed. Intergroup comparison of non-parametric variables was performed by Mann-Whitney U statistic test, and the paired Wilcoxon signed-rank test was used for intragroup analysis. Spearman’s correlation analysis was conducted to examine the association among the postoperative sizes of EZ and ELM defect, the CFT and the postoperative 12-month CDVA in both groups.

Linear regression analysis examined the correlation between morphological and clinical parameters (independent variables) and postoperative CDVA (dependent variable) in multiple regression model.

Data were analyzed using IBM SPSS Software version 22.0 (SPSS, Inc., Chicago, IL, USA). All *p*-values were two-sided and *p*-values ≤ 0.05 were considered statistically significant.

## 3. Results

Forty-three patients initially entered in the study. Three patients withdrew before randomization for personal reasons leaving 40 patients enrolled, but 1 patient withdrew during follow-up due to inconstancy, leaving a total of 39 patients (39 eyes) available for analysis (Table 1). Nineteen patients (19 eyes) were included in the IFCG-stained group and 20 patients (20 eyes) were included in the BBG-stained group. The two groups did not differ significantly in terms of the following parameters: mean age (range 52–84 years), duration of disease (range 2–15 months), minimum FTMH diameter (range 400–860 µm), base FTMH diameter (range 805–1355 µm). Eight eyes in the IFCG group and six in the BBG group were phakic with variable degree of cataract, and the mean preoperative CDVA did not differ significantly between groups (range 1.3–0.4 logMAR units), with a Snellen-equivalent acuity not above 20/123.

CDVA improved postoperatively in both groups at both 6 and 12 months (Table 2; *p* < 0.0005). The postoperative 6-month logMAR CDVA was 0.49 (20/61 Snellen equivalent) in IFCG and 0.39 (20/49 Snellen) in BBG eyes, a non-significant difference, while at 12 months a significantly worse CDVA in IFCG vs. BBG group was found (0.46 and 0.27 respectively, *p* = 0.036). No difference was found in terms of number of eyes with improved/stable CDVA between groups at the endpoint (*p* = 0.231), and with a useful CDVA of 0.3 logMAR (20/40 Snellen) or better present in 42% of IFCG subjects vs. 70% of BBG subjects (*p* = 0.111).

**Figure 1:**
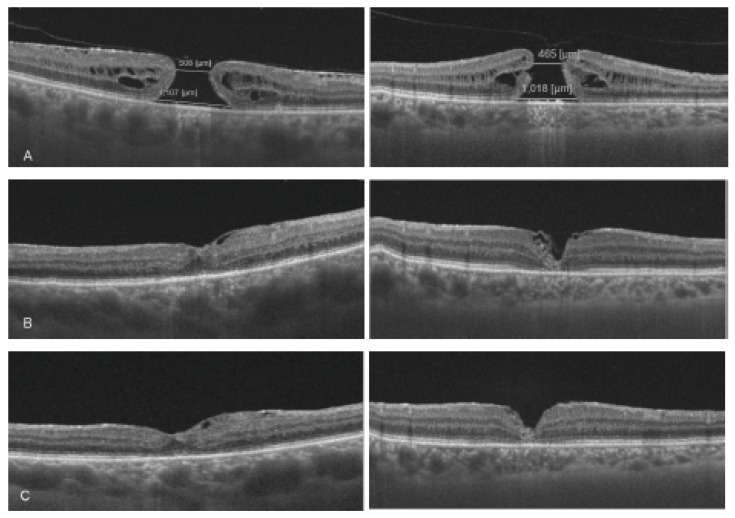
Swept-source OCT (SS-OCT) images of two cases before and after inverted flap surgery (I-ILM) for full thickness macular hole (FTMH). Left column: 65-year-old woman with 5-month FTMH since first diagnosis. Infracyanine green dyeing. Right column: 72-year-old man with 8-month FTMH since first diagnosis. Brilliant blue G dyeing. (**A**) Preoperative SS-OCT; (**B**) Six months postoperative SS-OCT; (**C**) Twelve months postoperative SS-OCT.

Table 3 shows the foveal configuration of the hole closure, sizes and closure rate of EZ and ELM defects, and thicknesses of the various retinal layers by ETDRS grid (CFT, MT, GCL++, pRNFL) by dye group at the two follow-up time points. In all cases, a complete closure of the FTMH was found (Figure 1).

The 12-month postoperative foveal morphology in both groups included mainly U-shaped cases, followed by W-shaped and V-shaped ones, without difference between IFCG and BBG group (*p* = 0.501, *p* = 0.605, and *p* = 0.716, respectively). The mean size of EZ defects at 6 and 12 months postoperatively did not significantly differ between groups, being lowest at 12 months in the BBG group and with complete endpoint closure rate of 26.3% in IFCG and 35% in BBG eyes (*p* = 0.893, *p* = 0.503, and *p* = 0.810). The mean ELM defect after 6 and 12 months and the 12-month percentage of ELM closure, 37% and 45%, respectively, did not exhibit intergroup difference (*p* = 0.630, *p* = 0.294, and *p* = 0.847). Even if in BBG cases postoperative EZ/ELM defects were smaller and closure rate was higher than in IFCG, especially at 12 months, significance was not reached due to the wide standard deviation and the number of complete closure cases (defect = 0). In both groups the size of the 12-month ELM defect was significantly reduced with respect to the 6-month value (*p* < 0.01). The average 6-month CFT was equivalent in both groups (*p* = 0.347), and at 12 months it showed an increase in BBG eyes as compared to IFCG (*p* = 0.041).

**Figure 2:**
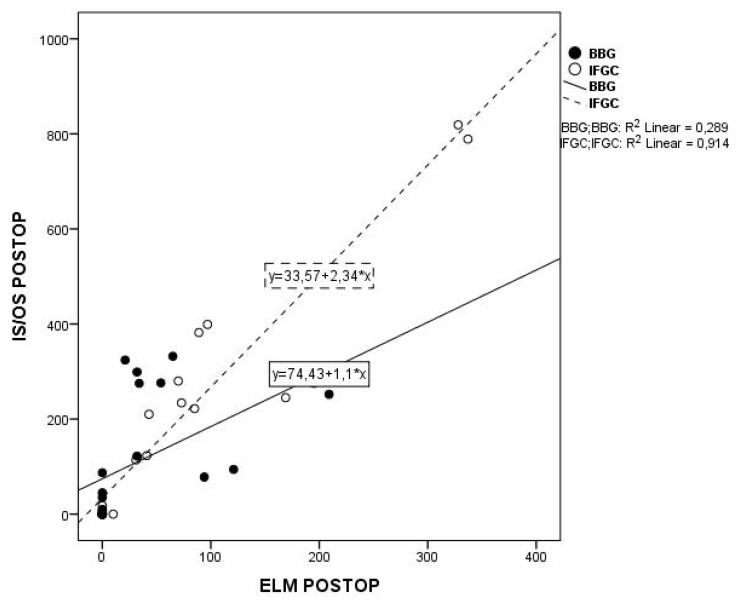
Lines of best fit through the 12-months. postoperative values of IS/OS (EZ) defect plotted against the ELM defect in IFCG (dashed line) and BBG (continuous line) group (R2 = 0.289 *p* < 0.0005 and R2 = 0.914 *p* < 0.0005). IS/OS = photoreceptor inner segment/outer segment junction (ellipsoid zone, EZ) ELM = external limiting membrane IFCG = infracyanine green BBG = brilliant blue G.

When comparing the 12-month postoperative sizes of ELM defect versus the EZ defect, a high correlation between the two parameters was found in both groups (*p* < 0.0005; Figure 2). Both 12-month EZ and ELM defects were inversely related to CFT (*p* = 0.002 and *p* = 0.001, respectively, in IFCG group and *p* = 0.003 and *p* = 0.001, respectively, in BBG group).

**Figure 3:**
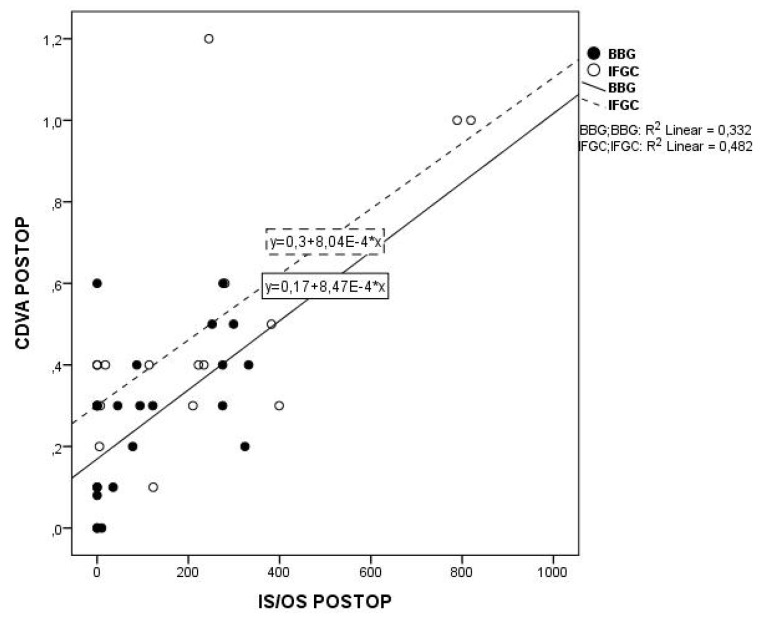
Lines of best fit through the 12 months. postoperative values of CDVA in log MAR plotted against the IS/OS (EZ) defect in IFCG (dashed line) and BBG (continuous line) group (R2 = 0.482, *p* = 0.012 and R2 = 0.332, *p* = 0.004 respectively). CDVA = corrected distance visual acuity IS/OS = photoreceptor inner segment/outer segment junction (ellipsoid zone, EZ) ELM = external limiting membrane IFCG = infracyanine green BBG = brilliant blue G.

**Figure 4:**
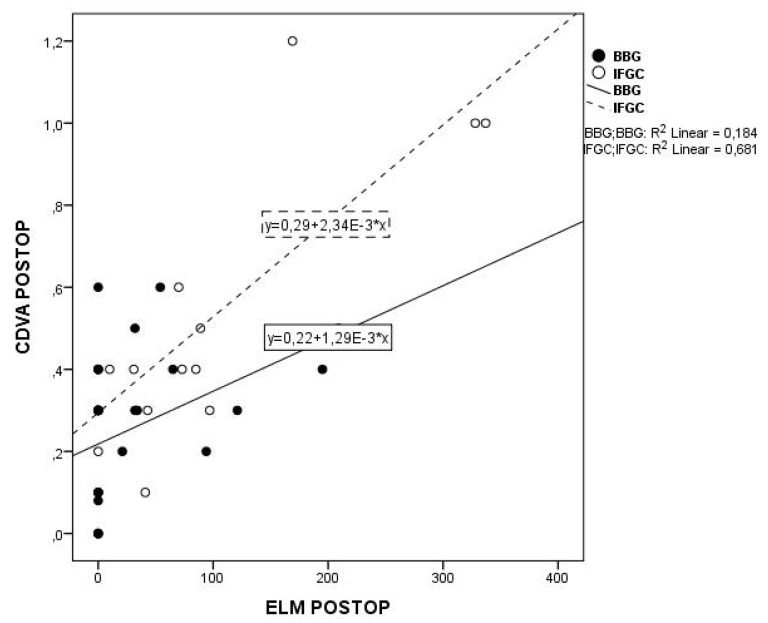
Lines of best fit through the 12 mo. postoperative values of CDVA in log MAR plotted against the ELM defect in IFCG (dashed line) and BBG (continuous line) group (R2 = 0.681, *p* = 0.0064 and R2 = 0.184, *p* = 0.013 respectively). CDVA = corrected distance visual acuity ELM = external limiting membrane IFCG = infracyanine green BBG = brilliant blue G.

The 12-month postoperative CDVA as a function of EZ and ELM defects in both groups is plotted in Figure 3 and Figure 4: it was inversely related to the EZ and to the ELM defect in both IFCG eyes (*p* = 0.012 and *p* = 0.004, respectively) and BBG eyes (*p* = 0.006 and *p* = 0.013, respectively). No correlation was seen between the postoperative 12-month CDVA vs. the CFT in either group (*p* = 0.056 and *p* = 0.204, respectively). Finally, on multivariate analysis, postoperative 12-month CDVA was related to preoperative CDVA in both groups (*p* < 0.0001 and *p* = 0.044, respectively), and to the type of dye, in this case BBG (*p* < 0.0001).

Concerning parafoveal area thickness (Table 3), the 3-mm-wide MT evaluation at 6 and 12 months failed to show intergroup differences. At 12 months a significant decrease with respect to 6 months in most sectors was anyway present in both groups (*p* < 0.01).

The 6-month GCL++ sector thickness values of the inner 3-mm grid area ranged from 98.4 ± 15.5 µm in T3 to 112.1 ± 24.9 µm in N3 in IFCG eyes, and from 105.6 ± 15.5 µm in T3 to 118.0 ± 12.3 µm in N3 in BBG eyes, again without difference (*p* > 0.05 for all four sectors). A small yet significant thinning at 12-month was present in all sectors, without intergroup difference, with the exception of the S3 sector, where the IFCG thickness of 95.3 ± 15.1 µm was smaller than the corresponding BBG one (*p* = 0.036).

The outer 6-mm grid GCL++ analysis at 6 months showed similar thickness in the two groups in N6 and T6 sectors, whereas IFCG thickness was less than BBG in the S6 and I6 sectors (*p* = 0.039 and *p* = 0.027, respectively). The 12-month values showed intragroup thinning in all sectors (*p* < 0.01), again with IFCG thickness less than BBG in S6 and I6 sectors (*p* = 0.011 and *p* = 0.009, respectively). Moreover, even in the T6 sector, a greater thinning of borderline significance was present in IFCG eyes (*p* = 0.05). No intergroup differences were found comparing the four pRNFL sectors at 6 and 12 months (*p* > 0.05 for all four sectors), with a 12-month thinning (*p* < 0.01), except for the pS sector in the IFCG group and the pN sector in both groups.

## 4. Discussion

We used the classic I-ILMFS as described by Michalewska et al. [3,5,40], which involves gently massaging the circularly trimmed ILM over the macular hole from all sides until it is inverted. We avoided insertion inside the hole by fill-in technique, to reduce the strength of the unavoidable contact between the dyed ILM and the foveal RPE [41] and, therefore, the RPE dye phagocytosis, possibly more dangerous with IFCG, as stated in the introduction [18,25,26].

ICG, without prejudice to its toxicity to the retinal ganglion cells and RPE, has been reported to produce greater stiffness of the stained ILM, an enhanced separation of the ILM from the underlying retina, and a more discernible contrast to the human eye than BBG. These characteristics are shared with the less toxic IFCG [22,23,24,42,43,44].

Even recently, authors from eastern Asian countries report successful off-label use of ICG for FTMH surgery in high myopia with or without retinal detachment, sometimes due to unavailability of BBG, with immediate ICG washing and excess removal by suction, possibly preceded by protection of the FTMH area with ophthalmic viscoelastic device [4,6,34].

In our prospective study, two groups of eyes affected with large FTMHs were randomly treated by I-ILMFS with IFCG, regarded as less toxic than ICG, or BBG. The latter is currently the most widely used ILM dye in western countries, characterized by very low toxicity at the RPE level, even if the possibility of damage of the inner retinal layers cannot be completely excluded, as noted in the introduction. In our study, a 12-month postoperative endpoint was chosen due to the above-mentioned literature data on IFCG chronic toxic effect and to some studies claiming a long functional and anatomical recovery time after macular hole surgery [45].

The two groups of eyes, who were equivalent in terms of mean age, male/female ratio, disease duration, pseudophakic/phakic ratio, and preoperative CDVA, did not differ with respect to minimum and base hole diameter. As mentioned above, we chose to not measure the EZ and ELM preoperative defects, assuming them to approximate the base diameter of the hole, because the difficulties in identifying these layers while approaching the hole’s cuff in many of our cases, together with the variable degree of their vertical elevation along the hole walls, could make their measurement sometimes subjective. Data in the literature report that the preoperative EZ defect is usually wider than the FTMH’s base diameter, while the ELM defect is equivalent or smaller, but the great variability of the mean values among different authors may reflect the difficulty in measurement [29,37].

At 12-month follow-up, the majority of cases in both groups exhibited the physiological U-shaped foveal morphology, with a very few cases of W and V-shaped pattern, as similarly described in a recent study using I-ILMFS and SS-OCT analysis [37]. We did not evaluate CDVA separately in different closure types due to the small number of eyes with the latter two patterns. The optimal anatomical result is indirectly testified by the significant increase of mean CDVA at 6 months in both the IFCG and BBG group, without difference between them. Both groups showed a further increase in CDVA between months 6 and 12, but the significantly higher CDVA value in the BBG group could reflect a long-term IFCG toxicity. No differences between groups were found at the endpoint in number of eyes with improved or stable CDVA, nor in proportion of eyes with a useful <0.3 logMAR (i.e., ≥20/40 Snellen) CDVA. Previous studies on FTMH surgery by traditional simple ILM removal found significantly worse visual acuity and lower percentage of eyes with normal acuity after 3 to 6 months in ICG-stained vs. BBG-stained eyes [29,46]. In our cases, the late difference in CDVA between IFCG and BBG, without difference in improvement/stability, could therefore indicate a lighter and later IFCG toxic effect with respect to data available in the literature on ICG, despite that the I-ILMFS implies some degree of contact of the dyed ILM with the RPE, contributing to IFCG toxicity.

No difference could be found in terms of EZ/ELM defects and closure rate between groups, as detailed in the results section. In both groups, a significant reduction of the ELM defect from 6 to 12 months confirms the potential for long-term gradual recovery after I-ILMFS [45]. The higher value of the EZ vs. the ELM defect, which has been reported in other studies [29], could depend both on a larger preoperative defect, and therefore damage, or on the greater difficulty in recovery of a neuroepithelial cell layer, the EZ, with respect to a mainly connective one, the ELM. The average CFT, which was equivalent in the two groups at 6 months, increased from 6 to 12 months in BBG eyes, approaching normal macular thickness in healthy eyes by SD-OCT [47]. This finding could be interpreted as a better chance in BBG-dyed eyes to reach a physiological foveal structure recovery. Many studies employing standard SD-OCT have regarded the restoration of the EZ as representative of the reconstruction of photoreceptors, and therefore significantly correlated with visual function [48,49,50].

In this respect, a thinner central fovea has been regarded as an incomplete recovery of the outer nuclear layer and photoreceptors [48]. A close correlation among the above-mentioned parameters can be found in both groups in our study at the endpoint. In fact, EZ and ELM mean defect values are directly related, CFT and CDVA inversely correlated with EZ and ELM defect, and CDVA directly correlated with the preoperative value and with the use of BBG. On the contrary, the lack of correlation between CFT and CDVA, already reported in previous studies [29,37], could be also justified by the dependence of the CFT on the degree of glial cell proliferation facilitating the hole closure. All the above considerations rely on the possibility of chronic long-term toxicity of the IFCG phagocytosed by RPE cells, even if some authors failed to find any experimental or clinical sign of toxicity with the low IFCG concentration we used [21,22,23,24,51]. The higher water solubility would, on the contrary, allow for less intracellular penetration of BBG, despite the possible contact of the dyed inverted ILM with RPE during the first days under air/gas tamponade [22,24,37].

The substantial equivalence of the two groups with regard to the parafoveal macular area thickness (MT), which includes all the neural retina layers, and the inner 3-mm parafoveal area including ILM, GCL++ seems to deny difference in toxicity from the two dyes by SS-OCT evaluation at this level. The thinning of the outer 6-mm GCL++ area from 6 to 12 months in all sectors in both groups is in agreement with literature data, since, as described in the introduction, thinning of the inner retina, including GCL++ and RNFL, after ILM peeling has been described both with ICG and BBG in SD-OCT–based studies [24,30,31,32,33]. In our patients, we must at first note that in the majority of cases, as described in the surgical procedure section, ILM surgical manipulation began near the inferior or superior vascular arcade. This could justify the S6 and I6 sectors thinning of inner retinal layers found by SS-OCT in the IFCG group and could imply a better preservation of inner retina in the BBG group in spite of surgical trauma, in comparison with an impaired long-term recovery after localized trauma on IFCG-dyed feet of the Muller cells. The analysis of the pRNFL sectors thickness, in agreement with most of our results, failed to show any difference between the two groups. Once more, a 6-to-12-month thinning was present in the majority of sectors in both groups. The equivalence of thickness even in the peripapillary temporal sector (pT), which could be the most involved in dye toxicity and in surgical trauma, eventually implies undetectable differences in this area by SS-OCT.

A limitation of this study is the analysis of simple high contrast central acuity, without evaluation of contrast sensitivity and retinal sensitivity allowed by contrast acuity measurement and microperimetry. It is anyway to notice that MP-1 microperimetry failed to discern a difference during 12 months in one prospective study regarding two groups of patients dyed with IFCG vs. triamcinolone acetonide in traditional FTMH surgery [26]. These authors conclude that microperimetry is a psychophysical test that evaluates the function of the entire retina without differentiating the activity of a single retinal layer, and that it can be influenced by the visual cortex enhancement.

## 5. Conclusions

In conclusion, according to the literature, our study confirms the excellent results in terms of rate and morphology of large FTMH closure allowed by I-ILMFS enhanced by ILM dyeing. Furthermore, even the significant increase in CDVA in both groups testifies the adequacy of both IFCG and BBG to help the surgeon in performing this delicate type of surgery. Notwithstanding, our results on long-term CDVA, CFT, and GCL++ sectorial thinning seem to testify a slow progressive IFCG toxic effect when compared to BBG dye. This toxicity, even if possibly lighter and later with respect to data available in literature on ICG, pushes us to consider IFCG at best as a second choice compared to BBG, even in I-ILMFS, and support the use of BBG over IFCG.

## Figures and Tables

**Table 1:** Patient baseline characteristics.

	IFCG	BBG	*p*
No. of eyes	19	20	
Age	70.1 ± 7.1 years	67.8 ± 8.4 years	0.363 *
Sex (M/F)	7/12	9/11	0.747
Symptoms duration (Mo)	6.8 ± 3.3	7.1 ± 3.5	0.784 *
Minimum FTMH diameter	555.4 ± 129.8	604.5 ± 133.4	0.251 *
Base FTMH diameter	1002.3 ± 143.6	988.3 ± 202.9	0.806 *
Phakic/pseudophakic	8/11	6/14	0.514
PREOPCDVA (logMAR)	0.79 ± 0.23	0.89 ± 0.25	0.169 ^

IFCG = Infracyanine Green stained eyes; BBG = Brilliant Peel stained eyes; FTMH = full thickness macular hole; CDVA = Corrected distance visual acuity. * ANOVA test; ^ Mann-Whitney U statistic test.

**Table 2:** Postoperative visual acuity results.

	IFCG (n = 19)	BBG (n = 20)	*p*
CDVA (logMAR) 6months	0.49 ± 0.33 §	0.39 ± 0.19 §	0.748
12months	0.46 ± 0.29 §	0.27 ± 0.1 9§	0.036
12^th^ mo. CDVA: improved	17	20	0.231/Fisher test)
stable	2	0	
worsened	0	0	
Eyes with CDVA ≤ 0.3 log MAR (≥20/40 Snellen) at 12 mos.	8 (42%)	14 (70%)	0.111 (Fisher exact test)

IFCG = Infracyanine Green stained eyes; BBG = Brilliant Peel stained eyes; CDVA = Corrected distance visual acuity. § *p* < 0.0005 with respect to preop. CDVA in both groups.

**Table 3:** Six-months and twelve-month postoperative foveomacular morphology, foveomacular and peripapillary layers thickness.

	IFCG	BBG	*p* Intergroup (Mann-Whitney U Test)
12 months U-shaped closure	12 (63.1%)	15 (75%)	0.501
V-shaped closure	2 (10.5%)	1 (5%)	0.605
W-shaped closure	5 (26.3%)	4 (20%)	0.716
EZ Defect (µm) 6 months	206.8 ± 257.9	160.1 ± 170.4	0.893
12 months	202.5 ± 251.1	119.2 ± 130	0.503
ELM Defect (µm) 6months	74.3 ± 103.9	51.9 ± 67.9	0.630
12months	72.3 ± 102.7 *	40.8 ± 63.6 *	0.294
CFT (C1, µm) 6months	144.8 ± 43.7	152.4 ± 36.6	0.347
12 months	150.9 ± 40.5	174.2 ± 34.9 *	0.041
MT (µm): S3 6months	294.8 ± 18.5	292.6 ± 17.6	0.573
12 months	274.3 ± 27.5 *	290.1 ± 118.7 *	0.169
N3 6months	296.3 ± 23.8	300.0 ± 24.5	0.936
12 months	283.7 ± 22.8 *	294.6 ± 23.6 *	0.294
I3 6 months	290.8 ± 14.9	289.5 ± 21.0	0.979
12 months	279.9 ± 15.7 *	284.5 ± 19.6 *	0.361
T3 6 months	283.3 ± 29.1	281.8 ± 20.2	0.748
12 months	265.6 ± 28.9 *	280.4 ± 19.5	0.124
GCL++(µm):S3 6 months	107.3 ± 18.1	109.7 ± 13.9	0.810
12 months	95.3 ± 15.1 *	108.1 ± 13.8 *	0.036
N3 6 months	112.1 ± 24.9	118.0 ± 12.3	0.124
12 months	108.8 ± 22.1 *	116.6 ± 11.8 *	0.088
I3 6 months	108.6 ± 14.1	108.9 ± 11.6	0.708
12 months	104.8 ± 12.2 *	107.9 ± 11.2 *	0.270
T3 6 months	98.4 ± 15.5	105.6 ± 15.5	0.196
12 months	91,1 ± 13.5 *	100.5 ± 14.8*	0.057
S6 6 months	87.4 ± 8.4	92.5 ± 11.9	0.039
12 months	85.1 ± 7.8 *	91.9 ± 11.9 *	0.011
N6 6 months	104.4 ± 15.7	104.4 ± 16.1	0.573
12 months	100.9 ± 14.6 *	103.6 ± 16.1 *	0.915
I6 6 months	86.7 ± 10.6	96.7 ± 13.7	0.027
12 months	84.4 ± 9.9 *	95.6 ± 13.4 *	0.009
T6 6 months	75.7 ± 9.8	81.7 ± 12.1	0.130
12 months	73.0 ± 9.4 *	81.0 ± 12.0 *	0.050
pRNFL(µm): pS 6 months	110.9 ± 13.2	114.1 ± 18.8	0.390
12 months	109.8 ± 11.3	113.7 ± 18.6 *	0.477
pN 6 months	82.7 ± 9.9	76.0 ± 15.8	0.215
12mo	82.9 ± 9.9	75. 9 ± 15.9	0.109
pI 6 months	117.7 ± 14.4	116.9 ± 15.2	0.979
12mo	115.4 ± 14.4 *	115.8 ± 14.7 *	0.872
pT 6 months	63.5 ± 8.6	68.5 ± 11.3	0.111
12 months	62.2 ± 8.3 *	68.2 ± 11.0 *	0.069

IFCG = Infracyanine Green stained eyes; BBG = Brilliant Peel stained eyes; EZ (IS/OS) = Ellipsoid zone (photoreceptor inner segment/outer segment); ELM = external limiting membrane; CFT = central foveal thickness; MT = macular thickness; GCL++ = internal limiting membrane/ganglion cells plus inner plexiform layer thickness; pRNFL = peripapillary retinal nerve fiber layer thickness. x3 = ETDRS 3-mm grid sectors; x6 = ETDRS 6-mm grid sectors. Px = pRNFL sectors. * *p* < 0.01 with respect to 6 month; Related-samples Wilcoxon Signed rank test.
